# Exploring the Effectiveness and Challenges of Community Rehabilitation Service Programs for Children with Developmental Delays: A Qualitative Study from the Perspective of Early Intervention Service Providers in Taiwan

**DOI:** 10.3390/children11080999

**Published:** 2024-08-16

**Authors:** Shu-Jou Sun, Wei-Lin Wang, Wei-Lun Huang, Wei-Sho Ho

**Affiliations:** 1 Department of Special Education, National Tsing Hua University, No. 521, Nanda Rd., East District, Hsinchu City 300193, Taiwan; sjsun@mx.nthu.edu.tw (S.-J.S.); light820128@gmail.com (W.-L.W.); 2 Eden Social Welfare Foundation, No. 5, Ln. 7, Sanshu Rd., Sanxia District, New Taipei City 237012, Taiwan; 3 Department of Industrial Education and Technology, Bao-Shan Campus, National Changhua University of Education, No. 2, Shi-Da Rd., Changhua City 500208, Taiwan; u107075701@cmu.edu.tw; 4 Medical Affairs Office, National Taiwan University Hospital, No. 7, Zhongshan S. Rd., Zhongzheng District, Taipei City 100225, Taiwan; 5 NCUE Alumni Association, Jin-De Campus, National Changhua University of Education, No. 1, Jinde Rd., Changhua City 500207, Taiwan

**Keywords:** early intervention, community-based early intervention services program, early intervention professional staff

## Abstract

This qualitative study aimed to investigate the effectiveness of community rehabilitation programs for children with developmental delays from the perspective of early intervention service providers in Taiwan. Adopting a single-case experimental design (ABM design), this study examined the immediate and sustained effects of interventions on individualized goals during baseline, intervention, and maintenance phases. Additionally, data from interviews with parents, special education teachers, and other participants were collected to understand the challenges and improvement strategies of community rehabilitation programs. Results revealed that community rehabilitation programs for children with developmental delays exhibited both immediate and sustained effectiveness. Challenges faced by parents and professionals differed, with parents having less contact and communication with administrative systems, while professionals experienced more pronounced implementation difficulties during interventions. Moreover, strategies for improving community rehabilitation programs for children with developmental delays should vary for parents and professionals to address inconsistencies in attitudes and strategies among parents and administrative obstacles encountered by professionals.

## 1. Introduction

Children are integral to societal stability, each having specific tasks and corresponding abilities at different developmental stages. Early developmental tasks significantly influence later ones. Some children require additional assistance from parents or educators to complete tasks at their developmental stage or even preceding stages. Early intervention services for children with developmental delays play a crucial role in providing this assistance [1,2]. Developmental delays are a group of “neurodevelopmental disorders” characterized by impairments in personal, social, academic, and occupational functioning. The *Diagnostic and Statistical Manual of Mental Disorders* (DSM-5) specifically refers to developmental delay as global developmental delay (GDD), which refers to a significant delay in the development of two or more areas, including cognitive, motor, communication, social, and self-care skills [3]. In addition to being troubled by the characteristics of their children with developmental delays, parents also feel disappointed or rejected by the lack of feedback during parent–child interactions. The effectiveness of early intervention for children with developmental delays cannot be overstated. The literature both domestically and internationally demonstrates that early intervention not only enhances children’s development in areas such as motor skills, language, communication, daily living skills, and socio-emotional development but also reduces future educational expenses and societal costs. Furthermore, it alleviates family stress, improves intra-family interactions, and enhances family functionality [4,5,6,7,8,9,10,11,12].

Numerous studies have affirmed the efficacy of early intervention, with governments enacting regulations to underscore its importance and define implementation frameworks. In the United States, the Education for All Handicapped Children Act Amendments of 1986 (Public Law 99—457) mandated early intervention services for infants and toddlers aged zero to six with developmental delays or at high risk. It emphasized interdisciplinary collaboration and family-centered services [13]. The Individuals with Disabilities Education Improvement Act of 2004 (Public Law 118—446), revised in 2004, specified components of early intervention programs, including family training, counseling, home visits, professional team assessment and services, and the appointment of a service coordinator responsible for individualized family service plans and coordination among agencies and professionals [14]. For example, research by Wang (2013) found that early intervention programs significantly improve the development and behavior of most children and have more positive effects on parents’ psychological, educational and information help [15]; Ueda et al. (2015) and Poon et al. (2014) pointed out that (the Family Outcomes Survey) can be used to evaluate family outcomes and effectiveness indicators of early intervention, and most families can feel family outcomes and highly effective early intervention services [16,17]. Tollan et al. (2023) summarized 23 studies on the effectiveness of early intervention and pointed out that the current early intervention paradigm has expanded from focusing on the effectiveness of individual intervention to policy practice for the well-being of families and other professionals [11].

In Taiwan, the Special Education Act was amended in 2019, stipulating early intervention for children with disabilities starting from the age of two, integrating medical resources for rehabilitation, training, and therapy [18]. The Child and Youth Welfare and Rights Protection Act also mandates government agencies, public and private institutions, and organizations to provide necessary services and measures for children in need of protection, assistance, counseling, therapy, early intervention, disability rehabilitation, and special assistance. Local governments are required to establish integrated service mechanisms and encourage or commission private entities to establish early intervention systems for children with developmental delays [19]. These policies underscore the global emphasis on early intervention.

Community-based rehabilitation primarily follows individualized intervention assessments to meet the long-term needs of infants and toddlers with developmental delays. Research has shown that providing early intervention services in the community enhances access to necessary resources and support for children with developmental delays and their families. It also increases interaction and integration with the community, leading to positive improvements in the abilities and efficacy of both children and their families [20,21,22,23,24,25,26,27]. However, in Taiwan, the distribution of early intervention resources varies geographically, resulting in inadequate distribution and inconsistent quality. Families of children with developmental delays in remote areas face difficulties accessing these resources, often requiring significant time and transportation costs [28]. In response, in 2004, it formulated the “Implementation Plan for Home-based Services for Children with Developmental Delays”, which provides professional teams to deliver services directly to homes, thereby mitigating the challenges faced by families in remote areas who lack access to resources available in urban centers [29]. In 2013, the Ministry of Health and Welfare proposed the “Implementation Plan for Community-Based Rehabilitation Services for Children with Developmental Delays”, aiming to provide early intervention services closer to families living in remote areas, reducing their time and transportation costs [29,30]. This initiative was further integrated into the “Implementation Plan for Community-Based Rehabilitation Services for Children with Developmental Delays” in 2016, establishing service processes and assessment mechanisms. It involves professional teams, parents, or caregivers in developing individualized family service plans (IFSPs), social work service plans, or family service plans to address the early intervention needs of children [31]. However, implementing community rehabilitation faces various challenges, including transportation issues, difficulties in promoting local service centers, passive attitudes among local parents, and a shortage of professionals [32]. Thus, it aims to explore the effectiveness of early intervention community rehabilitation services, understand related implementation challenges, and propose recommendations for early intervention services for children with developmental delays in the community.

## 2. Literature Review

### 2.1. Service Models in Early Intervention

According to Bailey and Wolery (1992), early intervention refers to services provided by professionals to infants and toddlers aged zero to six with developmental delays and their families [33]. These services encompass education, therapy, prevention, and family support tailored to the diverse needs of each family. The aim is to enhance the developmental prospects of delayed children and assist family members in their growth and adaptability. Early access to medical, educational, and social welfare resources may mitigate or prevent the worsening of developmental delays in children. Implementing early intervention not only addresses the delays in infants and toddlers but also provides supportive services for their families, thereby reducing societal costs and upholding children’s rights [33,34,35].

The service models of early intervention can be classified into different settings such as hospitals, homes, and communities. Early intervention in medical institutions typically occurs when newborns undergo the Apgar test at birth. If there are clear indicators of suboptimal conditions, hospitals immediately initiate reporting procedures and provide urgent medical interventions such as resuscitation or intensified care. This underscores the advantage of medical institutions for early intervention. However, apart from rare cases requiring prolonged hospitalization, most infants with developmental delays primarily reside in their family environment. Therefore, enhancing the adaptive capacity of families with delayed infants should be a primary consideration for intervention. Intervention in medical institutions may lack sufficient influence on the family environment aspect except in cases requiring extended hospital stays [36].

Home-based early intervention programs, also known as “family-centered services,” emphasize meeting the needs of families and enhancing functionality to improve the efficacy of developmental interventions for infants with delays [37]. McBride et al. (1993) propose that family-centered services focus on the family, support and respect their decisions, and aim to enhance family functioning [38]. From a regulatory perspective in early intervention, the primary goal is to provide education to delayed infants in their most familiar environment, with parents serving as integral caregivers. Trained professionals conduct regular home visits to provide education and intervention, aiming to understand the needs and abilities of delayed infants [39].

Utilizing the community environment as the site for early intervention services underscores the importance of providing developmental interventions for infants with delays in natural settings. This approach integrates activities conducive to the development of delayed infants into their daily lives, aiming to enhance their behaviors and abilities [40]. Dunst and Bruder (2002) suggest that natural settings refer to places where children typically learn and develop daily life skills [37]. Apart from homes, communities or schools offer environments where children can maximize their potential with minimal constraints. Community-based intervention services are typically situated in daycare centers or relevant healthcare facilities within the community, such as health clinics or local hospitals. These services are structured within the welfare service system or are institutionally grounded in the community, integrating community resources to enhance the effectiveness of welfare services. Importantly, it ensures that infants with delays and their families are integral parts of the community, participating in shared educational, recreational, and community activities [41].

### 2.2. Community-Based Services for Developmentally Delayed Children

In the UK, a study on 45 children with autism spectrum disorder (ASD) aged four to five found significant language improvements after 12 months of community-based early intervention [42,43]. Similarly, research at the University of Rochester, involving 71 ASD-diagnosed infants, highlighted that interventions focusing on social engagement predicted better outcomes in IQ and social adaptive behavior [27,44]. At the University of Pennsylvania, a study involving 79 ASD-diagnosed infants demonstrated notable enhancements in language expression and cognitive abilities post-community-based early intervention [45].

Lin (2013) found that Miaoli County utilized a case service model to connect resources, meeting the long-term developmental needs of infants with developmental delays. They provided home-based intervention strategies, enhancing parental understanding of early intervention [20]. Lin (2013) highlighted in Taitung County the establishment of community centers offering stable intervention programs through multidisciplinary teams. They also formed a parent support network and conducted infant development screening, promoting awareness and increasing screening frequency by local authorities [20].

Wang (2007) explored the challenges faced by early intervention institutions, categorizing them into curriculum and teaching, professional collaboration, parental involvement, and administrative resources [46]. Gao and Lan (2020) identified issues in Taiwan’s home-based services, including manpower shortages, unregistered recipients, undefined standards, large service areas with transportation difficulties, and funding pressures [5]. Ko (2009) found problems with policy promotion, service frequency, assignment indicators, tendering, service gaps, personnel challenges, and parental reliance [47]. Hong (2014) noted transportation and time allocation issues, difficulties in service promotion, passive parental attitudes, and professional shortages in rural areas [32]. Lien (2020) categorized service dilemmas into family conflicts, insufficient professional foundations, and parental attitudes affecting participation [48]. Huang (2018) highlighted limited resources, time constraints, curriculum gaps, speech development concerns, and parental pressures [22]. The Delan Center for Intellectual Development (2003) discussed difficulties in personnel nurturing, parental perceptions, learning environments, household interference, aid management, safety, and team integration [49]. Guralnick (2020) proposed the construction of a community-based early intervention system to support early intervention services for children with developmental delays and their families [10]. It can be seen that research on the effectiveness of early intervention has expanded from focusing on the effects of early childhood intervention to child and family outcomes, which is consistent with the research of Tollan et al. (2023) [11].

## 3. Methodology

### 3.1. Research Structure

**Figure 1:**
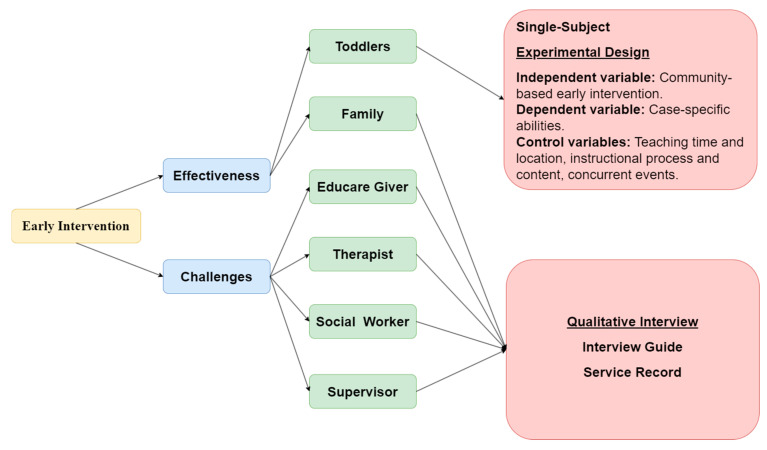
Research structure.

Due to the large individual differences between children with special needs and the small number of samples, it is not easy to meet the requirements of random assignment. However, a single-subject experimental design can achieve the experiment’s internal validity through individual internal comparison [50]. This study aims to investigate the effectiveness of community-based rehabilitation services for children with developmental delays from the perspective of early intervention service providers, employing the withdrawal design of a single-subject research method (as shown in Figure 1), including the baseline, treatment, and maintenance phases (referred to as ABM). Each subject received community-based rehabilitation services for three to four months, once a week for thirty minutes, for a total of twelve times. Secondly, the researchers only followed the daily teaching process in the baseline and maintenance phases without adding specific strategies. However, in the intervention phase, the researchers taught Participants A, B, and C through different strategies such as prompts and multisensory teaching to achieve their goals. Semi-structured interviews were conducted with parents, special education teachers, and other participants to gather data on the effectiveness and implementation challenges of early intervention community services.

### 3.2. Participants

This study adopted purposive sampling and selected children with developmental delays who participated in the New Taipei City Government’s community rehabilitation service program for children with developmental delays and obtained parental consent. The three children with developmental delays in this study all attend local kindergartens. Because they live in remote areas, it is difficult to obtain medical, special education, and social welfare resources in the community. Therefore, they are eligible to participate in the “New Taipei City Government Subsidy Community Rehabilitation Service Program for Children with Developmental Delays”. Community rehabilitation service centers provide early intervention services in local childcare centers or health clinics, which can integrate community-related resources and serve as the natural environment for children and family members’ daily life. Due to the shortage of therapist manpower, community rehabilitation service centers mainly provide special education and family support activities. Secondly, a total of 8 people were interviewed, including the mothers of 3 participants, 3 special education teachers and therapists, and 2 social workers and supervisors. The basic information of the participants is shown in Table 1.

#### 3.2.1. Participant A

A 5-year-old boy, the eldest child with a 6-month-old sister, diagnosed with mixed developmental delay and attention deficit/hyperactivity disorder (ADHD). Both parents are college graduates from an average socioeconomic background. The mother primarily accompanies the child for community rehabilitation services. Participant A exhibits moderate school performance but displays behavioral issues such as impulsivity and noncompliance with rules. He shows passive learning behavior and struggles with attention and concentration, particularly in group settings. Before the intervention, the evaluation results of Participant A in the “Infant and Toddler Early Intervention Curriculum: Assessment Manual” found that among the eight course evaluations, sensory perception ability performed best, while cognitive ability performed the worst. Since he was about to enter first grade, after discussing with his mother, we decided to use “writing his own name” in the cognitive domain as the intervention target.

#### 3.2.2. Participant B

A 4-year-8-month-old boy, the eldest child with a 2-and-a-half-year-old brother, diagnosed with global developmental delay and suspected language delay. His mother, originally from Mainland China, holds Taiwanese citizenship. His father is self-employed in construction-related design and is from a well-off background. The mother accompanies him for community rehabilitation services. Participant B has poor language expression abilities and primarily communicates by asking questions without responding. Before the intervention, the evaluation results of Participant B in the “Infant Early Intervention Curriculum: Assessment Manual” found that among the eight course evaluations, sensory perception ability performed best, while fine motor ability and communication ability performed the worst. Because Participant B has been receiving community intervention services for a long time, he could interact with the authors in appropriate sentences under the guidance of the authors. Considering that Participant B rarely engaged in pen-holding and pen-handling activities at home, after discussing with his mother, we decided to target “use the first three fingers to correctly hold a pen to draw or write” in the fine motor area.

#### 3.2.3. Participant C

A 4-year-5-month-old boy, the eldest child with a 2-year-old sister, diagnosed with mixed developmental delay. Both parents are college graduates and run a small bakery. The mother accompanies him for community rehabilitation services. Participant C struggles with language expression and often responds to questions by not answering or exhibiting negative behaviors. He shows limited vocabulary and difficulty forming complete sentences. However, with prolonged interaction, he can express basic needs. The mother also said that Participant C has better communication and expression skills at home, so the authors judged that he may have less interaction and speaking performance with strangers. Before the intervention, Participant C’s evaluation results in the “Infant and Toddler Early Intervention Curriculum: Assessment Manual” found that among the eight course evaluations, sensory perception ability performed best, while cognitive ability performed the worst. Since Participant C’s cognitive ability to number symbols was unstable, after discussing with his mother, we decided to use “can recognize more than two objects or pictures” in the cognitive area as the intervention target.

### 3.3. Research Tools

The present study utilized the “Infant and Toddler Early Intervention Curriculum Guidelines”, published by the First Social Welfare Foundation (2004), as a research tool. These guidelines outline the skills and behaviors necessary for infants and toddlers to develop independent abilities and cope with environmental demands [51]. The guidelines categorize developmental areas into eight domains: sensory perception, gross motor skills, fine motor skills, cognitive abilities, communication skills, social adaptation, activities of daily living, and leisure and recreation abilities. The criteria for these domains are clearly defined and easily understood by parents of developmentally delayed children, facilitating their comprehension. Moreover, the intervention requirements and goals during service delivery are primarily based on the child’s abilities at home, allowing the performance in the domains of activities of daily living and leisure activities to reflect the child’s relevant abilities in the home environment.

This study drew upon the relevant domestic and international literature, documents related to early intervention community rehabilitation services, and the researchers’ own experiences in early intervention community services to develop the interview outline. The interview outline served as a guide for conducting interviews and was adjusted in a timely manner based on the responses of different interviewees and the specific circumstances at the time of the interview.

### 3.4. Data Analysis

The data processing of this study included quantitative data and qualitative data, using inter-rater reliability and visual analysis to understand the effectiveness of three subjects participating in community rehabilitation service programs. Qualitative data were summarized through the interview results of the interviewees to understand the difficulties and improvement strategies in the intervention service process. In terms of inter-rater reliability, both the researcher and the observers had substantial background knowledge regarding observation recording. During the baseline, experimental intervention, and maintenance phases, the researcher and observers utilized a retrospective method by recording observations via video playback independently to understand the consistency of the occurrence of the target behaviors. A higher percentage in the calculated results signifies greater consistency between observers and the researcher [52,53]. According to Kazdin, good inter-observer agreement should range between 80% and 100%, with consistency among observers not falling below 75% considering different observation targets or a larger number of categories [54]. In this study, during the baseline period, the inter-rater reliability among observers for Cases A, B, and C all reached 100%. During experimental intervention period, Case A’s observer reliability averaged around 99%. For Case B, observer reliability averaged about 95%. Case C’s observer reliability averaged approximately 99%. During the maintenance period, Case A’s observer reliability averaged around 97%. Case B’s observer reliability averaged about 95%. Case C’s observer reliability averaged approximately 99%.

Visual analysis, including within-phase and between-phase analyses, was employed in this study. Changes in the frequency of target behaviors were presented through visual analysis using data from functional assessments. Charts and graphs were utilized to better understand the variations in target behaviors. This method was supplemented by photographic tools to provide detailed records of key points (timestamps) when target behaviors occurred for each case and to analyze the performance of relevant target behaviors by plotting curves.

In terms of qualitative analysis, the authors compiled an interview outline to explore issues related to children with developmental delays receiving community early intervention services and obtained the consent of the research participants in advance using recording equipment to record the interview content. After the interview, the content was transcribed and organized into text as soon as possible, and the interviewees were asked to confirm the accuracy of the content. The authors used two methods, member checking and triangulation, to test the credibility of the qualitative research results [55,56]. In the process of data processing and interpretation, researchers jointly discussed and referred to the relevant literature on the following: “open coding”, “axial coding”, and “selective coding”. They first found common keywords or key events such as early intervention service advocacy, insufficient professional resources, and forming an open code. Then, the researchers compared and summarized the main concepts among different materials that formed the main axis [57,58,59]. Finally, the authors categorized, compared, and analyzed the data according to the research purpose and questions to obtain the research results.

## 4. Result

### 4.1. Assessing Intervention Outcomes for Developmentally Delayed Toddlers

#### 4.1.1. Participant A

**Figure 2:**
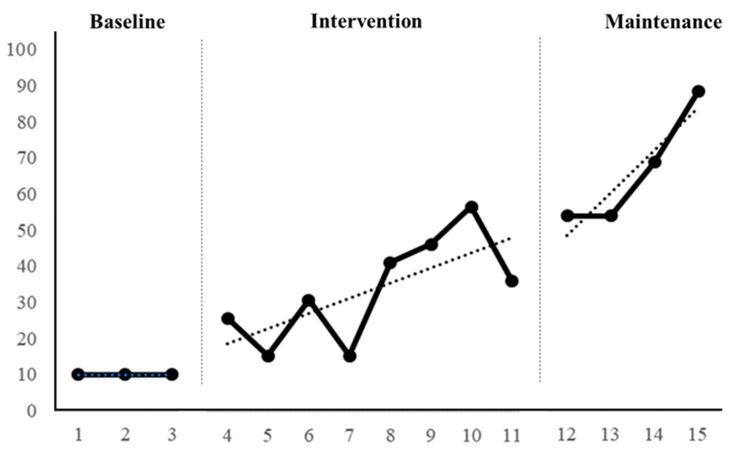
Participant A’s intervention line graph.

During the baseline period, Participant A could only perform basic writing strokes and lacked the concept of writing their own name. The range of their ability to write their name was 10 to 10, with no variation within the stage, averaging 10, indicating stability (100% stability). Participant A’s trend path for writing their name was “—”, with a stable trend (100% stability), leading to intervention. However, due to concurrent attention-related issues, the effectiveness of the intervention process was unstable. During the intervention period, Participant A’s ability to write their name ranged from 27.7 to 37.4, with a variation of 10 within the stage, averaging 32.5. The trend path for writing their name was “/”, showing an upward, positive trend but with instability (25% stability in level and 50% in trend). During the maintenance period, Participant A’s ability to write their name ranged from 55 to 90, with a variation of 35 within the stage, averaging 67.5, showing stability in trend (100% stability) but instability in level (25% stability). Refer to Figure 2 and Table 2 for details.

*Excerpts from relevant logs:*

“During the baseline period (1st, 2nd, 3rd sessions), Participant A could only perform basic writing strokes and simple symbol writing, such as circles and triangles. However, they did not possess the ability to write their name or related stroke concepts” (log entries A1101001, A1101002, A1101003).

“During the intervention period (5th, 7th sessions), Participant A exhibited poor attention during the intervention process, often appearing distracted. After discussion with the parent, it was determined that this might be due to inadequate mental alertness, as Participant A’s service time was from 14:30 to 15:30, and they usually napped at school until 15:00. It was concluded that Participant A might not have been fully awake” (log entries A1101102, A1101104).

“During the intervention period (11th session), Participant A showed poor attention during the intervention process. Therefore, the researcher engaged Participant A in relevant large motor activities before the writing intervention. However, Participant A’s attention could not be regained due to excessive excitement from the motor activities, resulting in poor performance during the writing intervention” (log entry A1101204).

“During the maintenance period (14th, 15th sessions), Participant A showed significant improvement in writing their name, achieving basic goals independently without researcher guidance. Although there were still reminders needed for certain aspects of font and spatial configuration, Participant A had achieved basic goals. It was noted that the parent also engaged in relevant exercises and activities with Participant A at home” (log entries A1110103, A1110104).

From the baseline to the intervention period, Participant A showed a level variation of 15 and an average level change of 22.5, with a positive trend direction and effect. There was no overlap (0% overlap), indicating positive progress in writing their name from baseline to intervention. From the intervention to the maintenance period, there was a level variation of 20 and an average level change of 35, with a positive trend direction and effect. There was a 50% overlap, indicating that despite the withdrawal of strategies, Participant A still maintained certain effectiveness, influenced by their performance during the intervention period. From the baseline to the maintenance period, there was a level variation of 45 and an average level change of 57.5, with a positive trend direction and effect. There was no overlap (0% overlap), indicating a significant improvement in Participant A’s ability to write their name. Refer to Table 3 for details.

#### 4.1.2. Participant B

**Figure 3:**
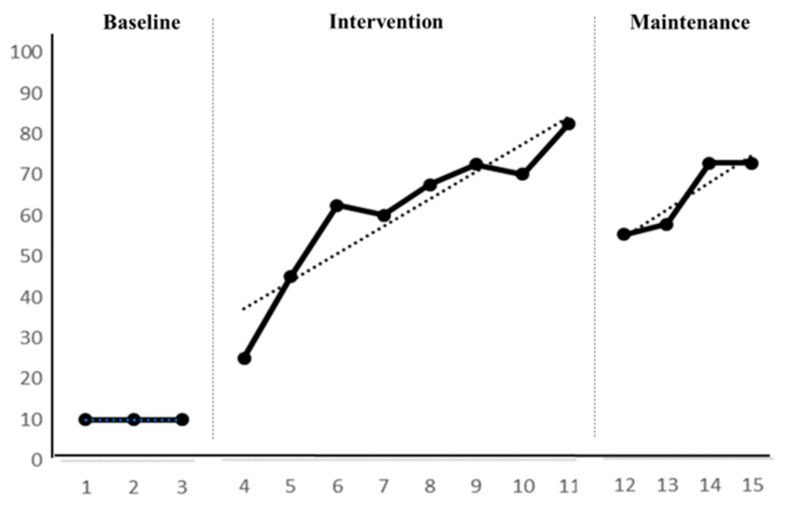
Participant B’s intervention line graph.

During the baseline period, Participant B exhibited a consistent level of using the first three fingers correctly for gripping and drawing or writing, with a range of 10 to 10. There was no variation within this stage, indicating stability (100% stability). Participant B’s trend path for this ability was “—”, showing a stable trend (100% stability), thus entering the intervention period. During the baseline, Participant B could only compensate by using four fingers and struggled to control coloring, unable to complete related writing activities. In the intervention period, Participant B’s ability to use the first three fingers correctly for gripping and drawing or writing ranged from 25 to 82.5, with a variation of 57.5 within the stage and an average level of 60.6. The trend path for this ability was “/”, indicating an upward, positive trend but with instability (37.5% stability in level and 87.5% in trend). During the maintenance period, Participant B’s ability level ranged from 55 to 72.5, with a variation of 17.5 within the stage and an average level of 64.4, showing stability (100% stability) in both level and trend. Refer to Figure 3 and Table 4 for details.

*Excerpts from relevant logs:*

“During the baseline period (1st, 2nd, 3rd sessions), Participant B could only use the first four fingers for gripping. During this period, Participant B’s grip style involved using the ring finger as compensation, unable to solely use the first three fingers for relevant coloring or writing activities. Additionally, there were fewer gripping exercises with the participant at home” (log entries A1101004, A1101005, A1101006).

“During the intervention period (6th session), Participant B showed a significant reduction in using the first four fingers for gripping. However, despite using a thicker triangular pencil during the intervention, Participant B still couldn’t write or color smoothly and relied on compensation. The intervention mainly focused on motor skills” (log entry A1101107).

“During the intervention period (7th session), Participant B expressed discomfort holding the triangular pencil during the intervention and requested a different type of pen. As a result, the researcher provided a triangular-shaped colored pen for Participant B’s use. However, there was an increase in the number of times Participant B resorted to using the first four fingers for gripping that day” (log entry A1101108).

“During the intervention period (10th session), Participant B’s mental state was unstable, likely due to the emotional state of the parent that week. The parent had conflicts with the grandparent, and the parent explained many of these situations and reasons to the researcher on the intervention day, suggesting possible factors affecting Participant B’s emotional and mental state” (log entry A1101207).

“During the maintenance period (12th, 13th sessions), as the maintenance of their condition was not as expected by the researchers, inquiries were made to the mother about whether there were any related writing or coloring exercises conducted with Participant B at home. The mother stated that there were fewer exercises such as pen-holding and writing practiced at home, attributing the actual reasons to her belief that other daily activities were already aiding Participant B in improving fine motor skills and that additional practice in pen-holding was unnecessary” (entries 1110105, 1110106).

Participant B exhibited a level change of 15 from baseline to intervention, with an average change of 50.6. The trend direction and effect were positive, with no overlap, indicating a favorable progression in Participant B’s ability to correctly grip the pen with the first three fingers for drawing or writing from baseline to intervention. From intervention to maintenance, there was a level change of −27.5, with an average change of 3.8. The trend direction and effect were negative, with a 100% overlap, suggesting that despite relevant intervention, Participant B still retained some effectiveness even after the strategy was withdrawn. From baseline to maintenance, there was a level change of 45, with an average change of 54.4. The trend direction and effect were positive, with no overlap, indicating an improvement in Participant B’s ability to correctly grip the pen with the first three fingers for drawing or writing. Details are provided in Table 5.

#### 4.1.3. Participant C

**Figure 4:**
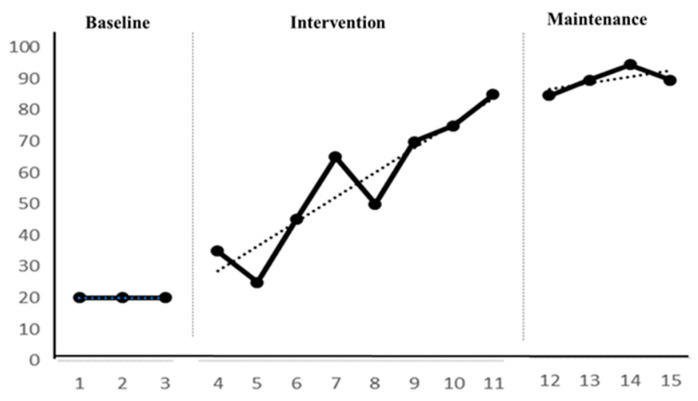
Participant C’s intervention line graph.

Participant C demonstrated a range of 20 to 20 in the ability to recognize whether two or more items/cards are the same or different during the baseline period. There was no level change within the phase, with an average level of 20, indicating stability with a stability rate of 100%. As the trend path for the ability to recognize whether two or more items/cards are the same or different was “—”, with a trend stability of 100%, it presented a stable state and thus progressed to the intervention period. During the baseline period, Participant C’s ability to recognize numerical symbols was unstable, with instances of digit reversal or writing reversal, particularly with the digits 2, 3, 5, 6, 8, and 9. In the intervention period, Participant C exhibited a range of 25 to 85 in the ability to recognize whether two or more items/cards are the same or different. There was a level change of 60 within the phase, with an average level of 56.3. The trend path for this ability was “/”, showing an upward, positive trend, and an unstable state (level stability of 25%, trend stability of 75%). During the maintenance period, Participant C’s ability to recognize whether two or more items/cards are the same or different ranged from 85 to 95. There was a level change of 10 within the phase, with an average level of 90 and a stability rate of 100%. The trend path remained “/”, with a trend stability of 100%, indicating a stable state. Details are illustrated in Figure 4 and Table 6.

*Excerpts from relevant logs:*

“During the baseline period (1st, 2nd, 3rd sessions), Participant C demonstrated satisfactory writing of symbols only for 1 and 4, with no reversals and the ability to consistently name the symbols correctly. Symbol 7 could be named correctly but occasionally had writing reversals, while symbols 2, 3, 5, 6, 8, and 9 showed poor writing conditions and difficulty in naming the symbols accurately” (entries A1101007, A1101008, A1101009).

“In the intervention period (5th session), Participant C’s recognition of symbols 2, 3, 5, 6, and 9 remained unsatisfactory. Additionally, on the intervention day, a therapist accompanied the session for a semi-annual professional consultation. Participant C’s cooperation and expression were less satisfactory with the presence of a stranger: (entry A1101110).

“In the intervention period (8th session), inquiries were made about Participant C’s completion rate of symbol recognition games at home. The mother indicated nearly 100% accuracy at home, but Participant C’s completion rate was only 50% during the intervention. It was later discovered that some symbols on the toys were handwritten by the researcher, leading to inconsistencies in recognition, particularly with symbol 9. As a result, all symbol toys were subsequently provided without inconsistencies for continued practice” (entry A1101207).

“During the maintenance period (15th session), as the community rehabilitation service for Participant C was transitioning to another unit, the mother explained the service personnel changes to Participant C. Consequently, Participant C exhibited overall unstable emotions and self-control on the intervention day. However, after communication and explanation by the researcher, assuring Participant C of the availability of other toys brought by new professionals for play, Participant C’s emotional and self-control condition stabilized considerably” (entry A1110112).

Participant C exhibited a level change of 15 from baseline to intervention, with an average change of 26.3. The trend direction and effect were positive, with no overlap, indicating a positive progression in Participant C’s ability to recognize whether two or more items/cards are the same or different from baseline to intervention. From intervention to maintenance, there was no level change, with an average change of 33.7. The trend direction and effect were positive, with a 25% overlap, suggesting that Participant C’s maintenance effect in related exercises was satisfactory and could be stably maintained for approximately 4 weeks, achieving short-term maintenance effects. From baseline to maintenance, there was a level change of 65, with an average change of 70. The trend direction and effect were positive, with no overlap, indicating a significant improvement in Participant C’s ability to recognize whether two or more items/cards are the same or different. Details are provided in Table 7.

### 4.2. Dilemmas Faced by Parents in Participating in Community Rehabilitation Services

This section presents interviews conducted with the mothers of Participants A, B, and C (referred to as Mother A, Mother B, and Mother C, respectively), who are the primary caregivers participating in community rehabilitation services. The purpose of these interviews was to understand the actual dilemmas encountered by parents after participating in community rehabilitation, as well as parents’ expectations for strategies to improve related issues.

#### 4.2.1. Lack of Access to Relevant Rehabilitation Information

Parents in rural areas have a low rate of access to relevant rehabilitation information. They generally perceive a lack of early rehabilitation advocacy, and most of them only become aware of relevant rehabilitation services after their children are identified by school teachers as having issues and are brought to local health centers. Alternatively, parents may become aware of such services when schools use developmental screening tools and notify them of their child’s need for community rehabilitation services.

“At the beginning, the school teacher thought there was an issue with the child and informed us that there was a foundation here offering services. I think advocacy is quite important because there are some newcomers or those who have married here, information is indeed less than elsewhere”. (Interview—Mother A1110101.)

“When I took my child to get vaccinated at the health center, the nurse gave me a developmental screening questionnaire, so I knew it”. (Interview—Mother B1110102.)

“Before, the teacher and the lady from the health center mentioned it. (So I had no prior experience or involvement with related rehabilitation services.)” (Interview—Mother C1110103.)

#### 4.2.2. Parents’ Level of Participation

The cooperation level of most parents is high. However, in this study, the primary caregivers selected are all mothers, and all three mothers are either housewives or have assistance from other family members in their shops. Therefore, the overall level of time commitment and implementation is higher compared to other cases.

“I think I just followed the teacher’s instructions and then practiced with my child at home. Yes, practicing according to the teacher’s methods and discussing with the teacher how to help the child, and then at home”. (Interview—Mother A1110101.)

“At home, in the beginning, language development was slower, so I told him more stories, talked to him more, and as for other activities”. (Interview—Mother B1110102.)

“I reviewed what the teacher taught in class with him at home to make him more proficient”. (Interview—Mother C1110103.)

#### 4.2.3. Communication and Interaction with Professionals

Due to the prolonged interaction time between these three parents and the researchers, the researchers were better able to communicate and explain the children’s conditions to the parents. It was also observed that even if it was therapists or social workers who were not frequently seen, parents could clearly understand the communication and explanation of their child’s condition. There were no issues observed in the interaction abilities of other professionals with parents, and the main difficulty in interaction might arise with doctors who are seen only once a year for assessment.

“I think it’s quite suitable for the children. Moreover, I think the teacher (researcher) and the occupational therapist are quite professional, so the child has been gradually improving”. (Interview—Mother A1110101.)

“It shouldn’t be too difficult… The only difficulty is when going for assessments before, isn’t it? Is it a consultation? Only the description by the doctor made me feel less understandable, but the other assessment teachers, if you don’t understand, you can ask them, and their explanations are not as vague”. (Interview—Mother B1110102.)

“No. Because if it’s really too difficult, I would ask myself”. (Interview—Mother C1110103.)

#### 4.2.4. Low Consistency in Parenting Among Family Members

The three parents demonstrate a relatively high sensitivity to their children’s conditions and all agree that parental autonomy is an important aspect of participating in services. However, all three families live with grandparents or great-grandparents, leading to observed disparities in parenting consistency among family members. The grandparents or great-grandparents themselves exhibit differences in their understanding of parental education and the implementation of parenting strategies compared to the mothers.

“I think because it’s rural, there are more cases of cross-generational parenting. Sometimes grandparents are also unsure of what to do and need more advocacy or external resources”. (Interview—Mother A1110101.)

“Because if you want to persist alone, it’s really tiring. Actually, he really needs family assistance and support… because older generations tend to think differently. For example, if a child isn’t very active, they think it’s not a big deal”. (Interview—Mother B1110102.)

“Grandparents may stick to old ideas, saying it’s okay if children talk late, but to doctors, it’s not okay, they have what is known as a language problem”. (Interview—Mother C1110103.)

#### 4.2.5. Insufficient Professional Resources

The three parents believe that early rehabilitation advocacy and external resources in rural areas are still inadequate. They hope for increased participation from other professionals such as speech therapists, physical therapists, occupational therapists, and psychologists. Additionally, they advocate for more appropriate rehabilitation spaces.

“I think we could add some different courses, diverse courses, because children need interactive activities involving large movements, so I hope there are such courses and spaces”. (Interview—Mother A1110101.)

“I hope there can be dedicated rehabilitation classrooms because teachers can bring more hands-on tools, which are easier to carry, but children also need activities involving large movements. If there is a room where we can put those large tools, it would be much more convenient for you as teachers”. (Interview—Mother B1110102.)

“I hope there will be relevant professional therapists who don’t come once in a while. I hope you can come once a month, not just every six months, because some families really can’t go out, so I hope there will be more help from your side”. (Interview—Mother C1110103.)

Based on the interviews conducted in this study, all three parents believe that “parental involvement” and “communication and interaction with professionals” did not pose significant difficulties in participating in community rehabilitation intervention programs. Mother A mentioned more often during the interview the issues of “lack of rehabilitation information” and “insufficient rehabilitation resources”. Mother B demonstrated more noticeable concerns regarding “low consistency in parenting among family members”, while Mother C exhibited more pronounced concerns about both “low consistency in parenting among family members” and “insufficient rehabilitation resources”. The summarized challenges faced by parents in participating in community rehabilitation include the following: (1) lack of access to relevant early rehabilitation information leading to a lack of awareness of community rehabilitation resources; (2) disparities in parenting attitudes and strategies among family members, affecting the effectiveness of rehabilitation; (3) insufficient professional resources, including inadequate manpower and inadequate rehabilitation space. Strategies proposed by parents to address these challenges include the following: (1) strengthening early rehabilitation advocacy to reduce parental information gaps and providing correct rehabilitation concepts to older generations to mitigate disparities in family members’ concepts; (2) adjusting rehabilitation spaces and increasing the participation of relevant community rehabilitation professionals. The results of this study are similar to those of Huang (2018) [14]. However, because all three trial parents are housewives, the second item in the Huang (2018) study, “The time limit for working in dual-income families requires assistance from alternative manpower”, did not appear in this study [14]. Additionally, the oral expression of Participants A, B, and C had improved before this study, so the fourth item in the Huang (2018) study, “Expectations for unsatisfactory oral performance of children and seeking support from special education resources”, did not appear in this study [14]. The results of this study regarding “parental involvement” show that parents did not feel insufficient in this aspect, which differs from the results of Wang (2007), Hong (2014), Lien (2020), and the Delan Enlightenment Center (2003) [24,39,41,42]. The reason for this is that for the sake of completeness, the selected trial participants were cases with fewer absences or missed classes, resulting in higher participation rates.

### 4.3. Challenges Faced by Professionals Participating in Community Early Intervention Programs

In this study, interviews were conducted with relevant professionals, including educational aides, therapists, social workers, and supervisors, to understand the challenges encountered by professionals participating in community early intervention programs and their expectations for strategies to address these challenges.

#### 4.3.1. Impact of Utilization of Relevant Intervention Resources on Practical Operations

Professionals perceive that during the implementation of community early intervention, they are often constrained by predetermined intervention locations or venues. This limitation affects the content and quality of the intervention services provided by professionals and prevents them from utilizing other resources available in the community to provide developmentally appropriate training or strategies for children with developmental delays.

“Community intervention should provide practical strategies for daily life. However, due to limitations in the service environment, the interventions lack community relevance”. (Interview with Educational Aide 1110401.)

“Because in rural areas, the service spaces lack large educational toys. Previously, we had to bring our own materials. For example, if we wanted to assess climbing ability, it’s limited in this service space. It’s not feasible to move materials daily”. (Interview with Therapist 1110403.)

“For example, some autistic children are taught using picture cards, but later we found they couldn’t associate the cards with real objects. Since we’re already in the community, why can’t we step out? Today, we’re fixed to this spot. We can’t take the children elsewhere in the community for visits. Although it’s called community intervention, I’m restricted to this space”. (Interview with Social Worker 1110404.)

“In recent years, you may have heard about the emphasis on family-centered and community-based approaches, aiming for more integration for children. If a child has social interaction issues, why can’t we take them to the park to interact with other children there? Also, let’s have parents practice actual operations. We don’t have to spend the entire hour outside. I can spend half an hour at home and half an hour outside. I hope this part can be more flexible for us”. (Interview with Social Worker 1110404.)

“In the past, there was a case with many children at home and limited financial resources. The child didn’t understand pretend play. So, during the assessment at kindergarten, the teacher gave him a toy cake, and he started eating it directly because he thought it was real. Due to the lack of such experiences, his abilities appeared severely delayed during the assessment”. (Interview with Supervisor 1110405.)

#### 4.3.2. Parental Involvement and Self-Concept Awareness

Professionals also perceive that during community early intervention, parents themselves often lack an understanding of the concept of community intervention services. This lack of awareness may lead to excessive concern or insufficient awareness. Factors such as low parental involvement also contribute to this situation. Additionally, parents still perceive community intervention as a task for professionals rather than something they can directly execute, which affects the implementation and effectiveness of related strategies.

“In rural areas, most elders focus on transportation and daily care. Therefore, requiring home exercises can be challenging. Also, due to the regional and cultural factors, parents may not prioritize or participate in interventions. For them, as long as they provide food and clothing, it’s enough. Asking them to do more can be really difficult. Additionally, some may have difficulty understanding written instructions. Therefore, we offer practical suggestions, such as teaching elders to teach children to put shoes and bags in fixed places”. (Interview with Educational Aide 1110401.)

“I think most cases I encounter require more intervention resources. At least, the ones I’ve dealt with do. Usually, it’s those who seem to need assistance more, like those in the middle- to low-income bracket. We often need to use some techniques to communicate with them… I think some parents also need to accept additional resources”. (Interview with Therapist 1110402.)

“Currently, many parents still don’t understand their child’s situation or are unaware of it. I think raising awareness on this aspect is necessary. Even some realize it too late”. (Interview with Therapist 1110403.)

“More often, it’s ‘do for them’ rather than ‘do with them.’ It shouldn’t always be us doing everything for them; instead, we should facilitate their involvement. But parents still perceive our intervention model as needing professionals. They need to realize they should be more independent, rather than us doing everything for them. I feel they just want the service, not the parenting aspect”. (Interview with Therapist 1110403.)

“Most parents, when faced with early intervention issues, often blame themselves first. Their guilty conscience makes them want the best for their children, willing to spend money, willing to resign from work, just to prevent developmental delays, not wanting another assessment report. Most parents get stuck in this fixation…What they can’t see is that early intervention is just a process at this point in time. Even if the child’s gross motor skills are poor today, it doesn’t mean something terrible will happen to them when they grow up”. (Interview with Social Worker 1110404.)

“Parents why do they want clinic-based interventions today? It’s because they can’t do it at home, and there are issues. Having issues means they need professionals. Parents think that I’m not as professional, so giving it to professionals is definitely not a problem. But I think clinic-based interventions don’t really help parents; I think they help parents improve their children’s abilities. But in terms of parental competence improvement is lacking”. (Interview with Social Worker 1110404.)

“Sometimes, when renting venues in rural areas, it’s still necessary to do more publicity because some elders are not familiar with early intervention. They may say there are no children here, only old people”. (Interview with Supervisor 1110405.)

#### 4.3.3. Human Resources Time and Expenses for Professional Personnel

Professional personnel also perceive a lack of sufficient resources in terms of manpower for actual execution. Moreover, since many cases are located in remote areas, the transportation time and mental state of professional personnel also affect the quality of service. Additionally, due to the unstable case volume, it is difficult to balance the time coordination and cost arrangement for part-time professional personnel.

“For some older teachers, their physical strength is limited. Unlike other units that have drivers, I feel that doing three cases in a day plus driving is already the limit. Having a fixed schedule might be better, but long hours of driving really affect mental well-being”. (Interview—Educational Counselor 1110401.)

“We often visit individual case families, but their problems may not be limited to just one. It may not only involve early intervention issues for children, but they may also require additional professional interventions, such as additional medical examinations or further tracking of specific conditions. I feel that some parents themselves also need to access other resources. At this point, assistance might be relatively limited from above, and I can only provide my own expertise to the parts that this child needs consistently. They may need other therapists or other professionals to come in, but what we can offer is only a single profession”. (Interview—Therapist 1110402.)

“The differences between regions are too large. In urban areas, children’s therapy sessions may be packed, but in rural areas, there is nothing available, and there is no choice in professionals”. (Interview—Therapist 1110402.)

“But from the perspective of practitioners, usually, the service messages I receive are short-term. The frequency is low, occasionally going once, and the frequency is not high. The case volume may not be high either. It’s just going to distant places and dealing with two or three cases like this. Otherwise, I have to work from Monday to Friday, and I can only work on Saturdays and Sundays. So, I have to take extra leave to serve the community, which I think is difficult”. (Interview—Therapist 1110402.)

“Suppose I go to other places, like the rural areas we went to before. In one morning, there may be two or three cases, or maybe three or four cases for the whole day, right? Then I have to take a day off inevitably, and subtracting the travel time, everyone has travel time to work. For that whole day, I can only serve these few children, and then it’s the next time, maybe it’s already the next semester, or even next year, or even later. Basically, I think the incentive for this is very low”. (Interview—Therapist 1110402.)

“Because I think if it’s once every six months for (professional consultation), I may still be getting to know the parents. So, there may be many things they (parents) may not necessarily mention. So, if this child really needs therapeutic activities, there may be some limitations, and some children may not need continuous (therapy). Some I may have encountered two or three times or more, and some may have only encountered once. I think it’s like meeting by chance, it’s hard to give them some advice on information”. (Interview—Therapist 1110403.)

#### 4.3.4. Communication and Processes with Administrative Systems

Professional personnel also perceive discrepancies between relevant administrative regulations and their practical execution. Moreover, there are often gaps in the transmission of relevant information, which subsequently affects the frontline professionals’ ability to adapt and respond in service delivery.

“However, after serving for such a long time, I have been thinking, shouldn’t the exercises the child does at school also count as part of his development practice? Why should he take a four-hour leave just for a one-hour session with us? Due to the current epidemic situation and the child’s daily routine, usually, when he needs to use our scheduled service, it’s usually based on taking a four-hour leave as a benchmark. It’s not just four hours in the morning; it could also be in the afternoon. So, if some children actually make stable progress through training at school, do they really need to take an hour, or rather, four hours off to receive a one-hour scheduled service? This is what I’m pondering (or community therapy resources)”. (Interview—Social Worker 1110404.)

“The related bureaucratic hierarchy within the administrative system is more likely to cause delays in time, whether in administrative processing or in the service delivery of frontline professionals. If the administration gets stuck, subsequent services will also be affected, such as the handover at the beginning of the year… Although we all know that the administrative system needs to go through layers of approval, because it is too bureaucratic, it leads to the inability to complete subsequent related tasks”. (Interview—Supervisor 1110405.)

“For example, in the case of fund disbursement operations, the administrative system may not complete the process, resulting in the inability to disburse funds. While this may be manageable for large units, smaller units may not necessarily have sufficient funding allocation for frontline professional personnel. The only thing units can do is to communicate with the administrative system, but this situation will affect the execution of the entire service plan”. (Interview—Supervisor 1110405.)

“I feel that sometimes there is a sense of communication breakdown, communication is not smooth (emphasis). When we ask questions (to the administrative system), it’s always about the difficulties we encounter in our work, and these are difficulties that we cannot solve on our own, and we rely on you. Like recently, with the epidemic getting worse. We ask, because some children are facing school closures and so on, is the epidemic getting worse? Should we just switch everything to online, or what measures should we take? Or can you tell us what indicators we should follow before we switch everything to online? However, they don’t respond, even though the epidemic situation is severe, and things are changing rapidly”. (Interview—Supervisor 1110405.)

After analyzing the interview data of this study, several professionals faced different challenges after participating in community intervention programs. The reasons for these different challenges are attributed to the diverse nature of their work roles. Teachers and therapists, who often interact directly with parents and children with developmental delays, emphasized issues such as the “impact of resource utilization on actual operations”, “parents’ level of involvement and self-concept awareness”, and “personnel time and budget constraints” as significant challenges affecting the implementation of community intervention programs. On the other hand, social workers and supervisors, primarily involved in backend coordination and oversight, identified challenges such as “the impact of resource utilization on actual operations”, “parents’ level of involvement and self-concept awareness”, and “communication and processes with administrative systems” as more pertinent to the operation and maintenance of backend community intervention programs.

The current challenges faced by professionals in community intervention include the following: (1) restrictions on resource utilization affecting actual operations and consequently the effectiveness of interventions; (2) lack of parental involvement and self-concept awareness, hindering the implementation and operation of relevant strategies; (3) difficulty in balancing personnel time and budget, resulting in a shortage of professional manpower; and (4) inflexible administrative processes and communication barriers, affecting the difficulties in related processes and services. Professionals anticipate strategies to alleviate these challenges, including strengthening advocacy for early intervention to reduce parental information gaps and providing practical parenting strategies for daily implementation, thus enhancing parental autonomy in community intervention involvement. They also advocate for relaxation of intervention space limitations to enable professionals to effectively utilize community facilities, fostering greater integration between children with developmental delays and the community. Additionally, increasing the quotas and budgets for relevant professionals is suggested to address the shortage of professional manpower. Furthermore, adjustments in administrative communication processes are proposed to ensure that executing units receive accurate information, thereby reducing service interruptions.

These identified challenges align with findings from previous studies. However, it is noted that the challenge of “restrictions on resource utilization affecting actual operations and consequently the effectiveness of interventions” bears a resemblance to the findings of Wang (2007) regarding “curriculum and teaching”, though it is not entirely identical. This study additionally highlights the differences between actual execution spaces and community spaces within this challenge [46]. The challenge of “lack of parental involvement and self-concept awareness, hindering the implementation and operation of relevant strategies” aligns with the findings of various other studies by Wang (2007), Ko (2009), Hung (2014), Lien (2020), Huang (2018), and the Delan Enlightenment Center (2003) [22,32,46,47,48,49]. Similarly, the challenge of “difficulty in balancing personnel time and budget, resulting in a shortage of professional manpower” resonates with findings from Wang (2007), Ko (2009), Hung (2014), Lien (2020), Huang (2018), and the Delan Enlightenment Center (2003) [22,32,46,47,48,49]. Lastly, the challenge of “inflexible administrative processes and communication barriers, affecting the difficulties in related processes and services” aligns with findings from Wang (2007), and Ko (2009) [47,48].

## 5. Conclusions

The main purpose of this study is to investigate the individual ability changes of children with developmental delays after participating in a community rehabilitation service program, including immediate effects and maintenance effects. It also explores the challenges faced by parents and professionals participating in the community rehabilitation intervention program, as well as strategies for addressing these challenges.

From the results of this study, it was found that the overlap rate between Participants A, B, and C between the baseline period and the intervention period was 0%, and the trend changes were all positive. It can be seen that the results of this study indicate that the effectiveness of the community rehabilitation service program for children with developmental delays has both immediate and maintenance effects. All three participating children showed good immediate effects on their individual target abilities, including “writing their own name”, “correctly gripping the pen with the first three fingers for drawing or writing”, and “recognizing whether two or more items/cards are the same or different”. Additionally, these target abilities were also well maintained over time. However, the individual maintenance effects varied, influenced by subsequent practice within the family.

Challenges faced by parents in the community rehabilitation service program for delayed children include the following: (1) inadequate early intervention information and lack of awareness of community rehabilitation resources; (2) inconsistent parenting attitudes and strategies within the family affecting the effectiveness of rehabilitation; and (3) insufficient professional resources, inadequate manpower, and inadequate space. Challenges faced by professionals in the community rehabilitation program for delayed children include the following: (1) limitations in the utilization of relevant rehabilitation resources affecting practical operations; (2) lack of parental participation and self-concept awareness; (3) difficulty in balancing manpower time and funding for professionals; and (4) inflexibility and ineffective communication with administrative systems.

Recommendations from this study suggest strengthening early intervention advocacy for parents, adjusting rehabilitation spaces, and increasing the number of relevant community rehabilitation professionals to improve the situation. Measures to address the challenges faced by professionals include relaxing restrictions on rehabilitation spaces, increasing quotas and budgets for relevant professionals, and adjusting information communication processes within administrative systems.

## 6. Limitations

Firstly, this study only focused on children who participated in the New Taipei City Government’s community rehabilitation service program for children with developmental delays, and the results should not be extrapolated to other regions. Secondly, parent participation is a major boost in community rehabilitation service programs, but this study only explored the effectiveness of three children with developmental delays after receiving early intervention services and did not explore changes in parents’ parenting abilities. Moreover, participants in this study only used early intervention services in the community rehabilitation service program, and results could not be extrapolated to children with developmental delays who used the home-based early intervention service model.

## Figures and Tables

**Table 1:** Summary of participants’ basic information.

Participant	Gender	Age	Education Level	Occupation/Experience
Mother A	Female	34	Bachelor	Labor
Mother B	Female	35	Senior high school	Housewife
Mother C	Female	38	Senior high school	Housewife
Educational aide	Female	44	Master	17 years
Therapist	Male	28	Bachelor	4 years
Social worker	Female	31	Bachelor	9 years
Supervisor	Female	31	Bachelor	9 years

**Table 2:** Participant A’s analysis of intra-phase changes table.

Stage	A	B	M
Number	3	8	4
Trend path	—	/	/
Trend stability	100%	50%	100%
Average level	10	32.5	67.5
Level range	10–10	15–55	55–90
Level stability	100%	25%	25%
Level change	0	10	35

**Table 3:** Participant A’s analysis of inter-phase changes table.

Stage	A/B	B/M	A/M
Trend change	/	/	/
Trend stability	Stable–unstable	Unstable–unstable	Stable–stable
Level change	15	20	45
Average level Change	22.5	35	57.5
Overlap percentage	0%	50%	0%

**Table 4:** Participant B’s analysis of intra-phase changes table.

Stage	A	B	M
Number	3	8	4
Trend path	—	/	/
Trend stability	100%	87.5%	100%
Average level	10	60.6	64.4
Level range	10–10	25–82.5	55–72.5
Level stability	100%	37.5%	100%
Level change	0	57.5	17.5

**Table 5:** Participant B’s analysis of inter-phase changes table.

Stage	A/B	B/M	A/M
Trend change	/	\	/
Trend stability	Stable–unstable	Unstable–stable	Stable–stable
Level change	15	−27.5	45
Average level change	50.6	3.8	54.4
Overlap percentage	0%	100%	0%

**Table 6:** Participant C’s analysis of intra-phase changes table.

Stage	A	B	M
Number	3	8	4
Trend path	—	/	/
Trend stability	100%	75%	100%
Average level	20	56.3	90
Level range	20–20	25–85	85–95
Level stability	100%	25%	100%
Level change	0	60	10

**Table 7:** Participant C’s analysis of inter-phase changes table.

Stage	A/B	B/M	A/M
Trend change	/	/	/
Trend stability	Stable–unstable	Unstable–stable	Stable–stable
Level change	15	0	65
Average level Change	26.3	33.7	70
Overlap percentage	0%	25%	0%

## Data Availability

Data available on request due to restrictions regarding privacy, legal and ethical concerns. The data presented in this study are available on request from the corresponding author.
